# Supplementary material for: Lifetime stability of social traits in bottlenose dolphins
Source: Commun Biol. 2021 Jun 18;4:759. doi: 10.1038/s42003-021-02292-x (PMC8213821; doi:10.1038/s42003-021-02292-x)
Supplement: Supplementary file 2 — Supplementary Information [file 42003_2021_2292_MOESM2_ESM.pdf]

**Lifetime stability of social traits in bottlenose dolphins: Supplementary material**

**Supplementary Figure 1**. Plots of four highly repeatable measurements across the lifespan.

**1a**. Time spent alone across the lifespan, calculated as proportion of sightings alone (including dependent mother-calf pairs). Males spent significantly less time alone than females (post. mean = -0.197, 95% CI = -0.26--0.14). Calves and the two oldest adult categories spent more time alone than the other age categories (post.mean = 0.095, 95%CI = 0.05-0.15; p.m = CI 0.07, 0.01, 0.14; p.m = 0.13, CI = 0.01-0.23). Juveniles spent less time alone than other age categories (p.m = -0.06, CI = -0.10—0.01).

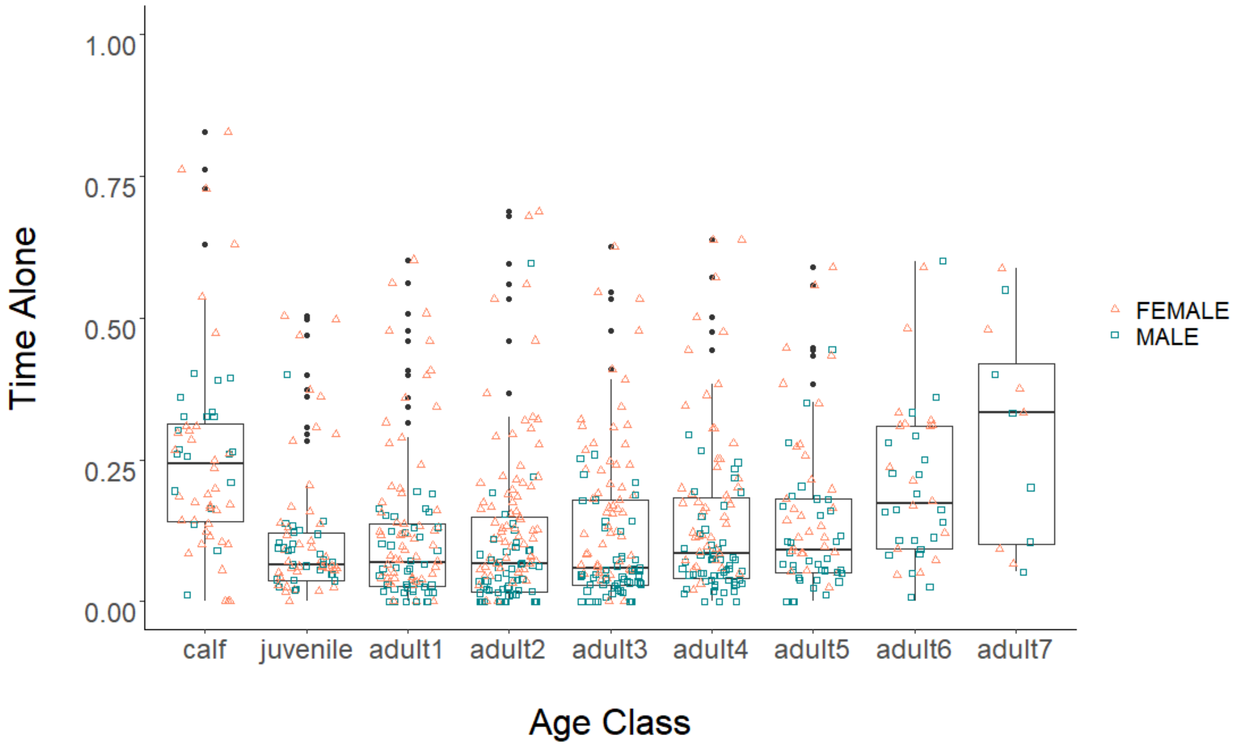


**1b**. Time spent in large groups (7 or more dolphins) across the lifespan. Males spent more time in large groups than females (p.m = 0.11, CI = 0.06-0.16). The two oldest adult categories spent less time in large groups than the rest of the age blocks (p.m = -0.07, CI = -0.12—0.02; p.m = -0.11, CI = -0.18—0.02).

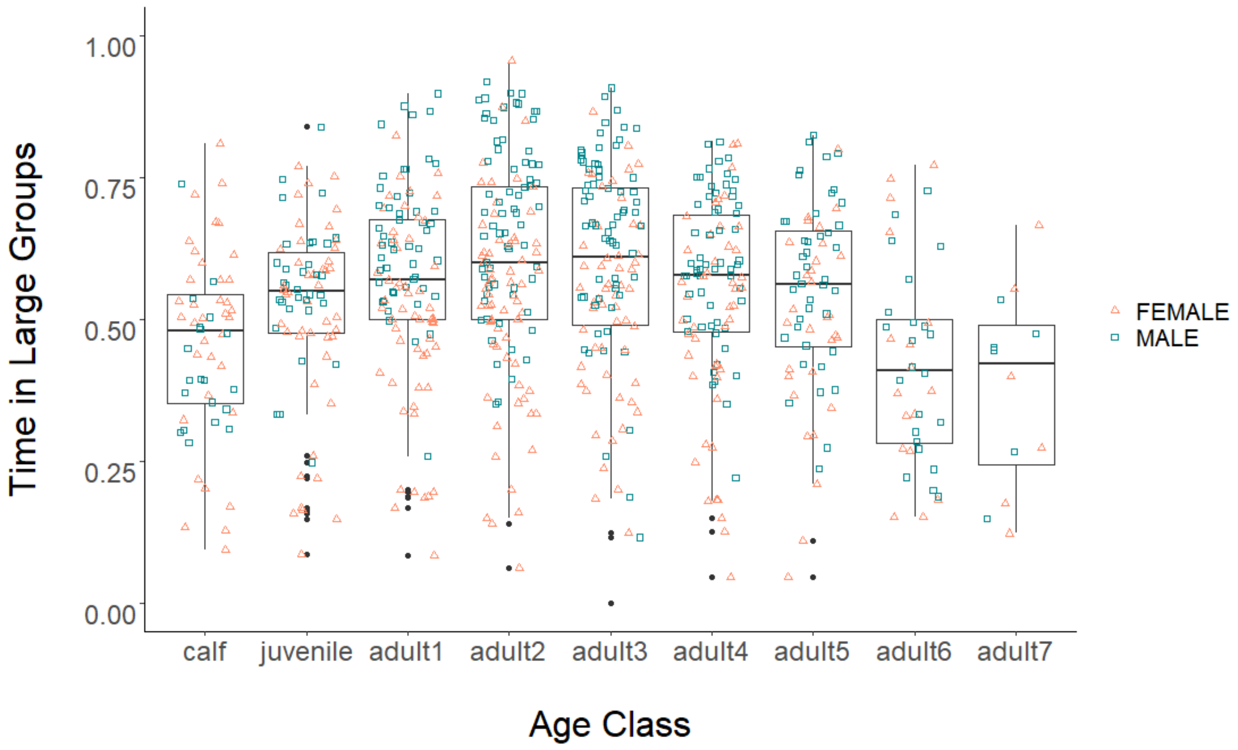


**1c**. Average number of associates across the lifespan. There was no significant effect of sex on the number of associates per individual. Calves and juveniles had fewer associates than other age categories (p.m = -9.6, CI = -13.35—5.53; p.m = -4.90, CI = -8.54—1.11).

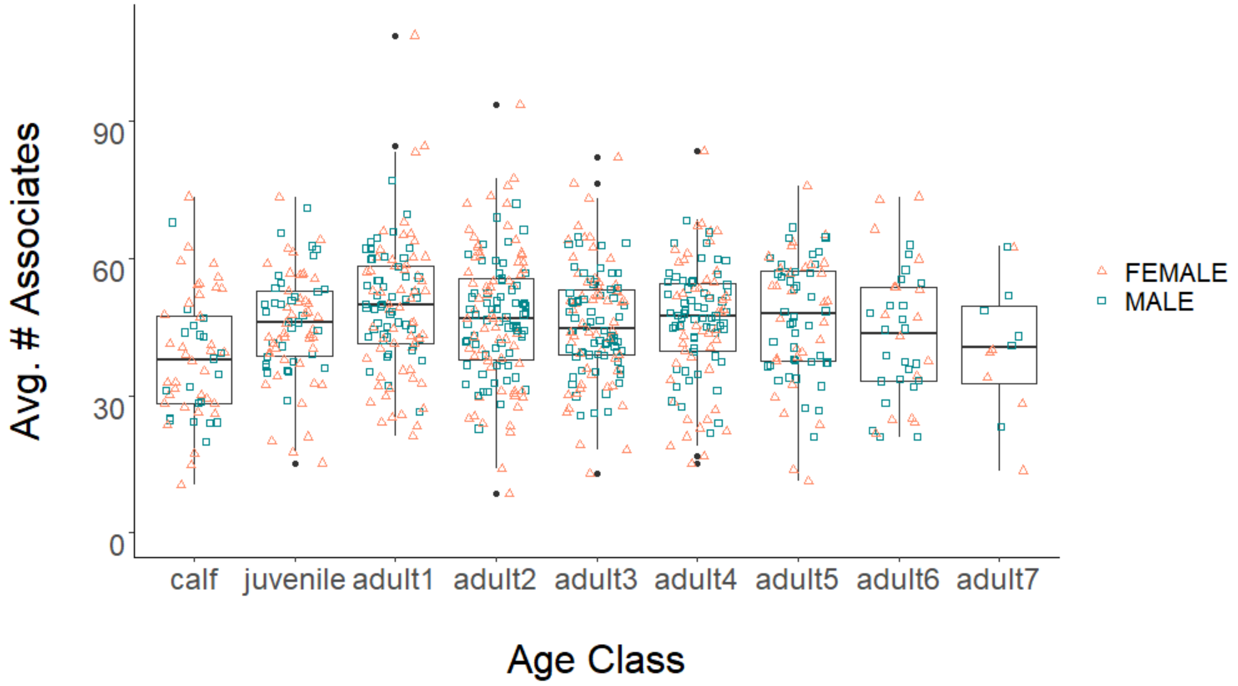

**1d**. Average number of same-sex associates across the lifespan. Males had more same-sex associates than females (p.m = 6.21, CI = 3.71-8.92). Calves had fewer same-sex associates than other age categories (p.m = -3.34, CI = -5.77—0.99).

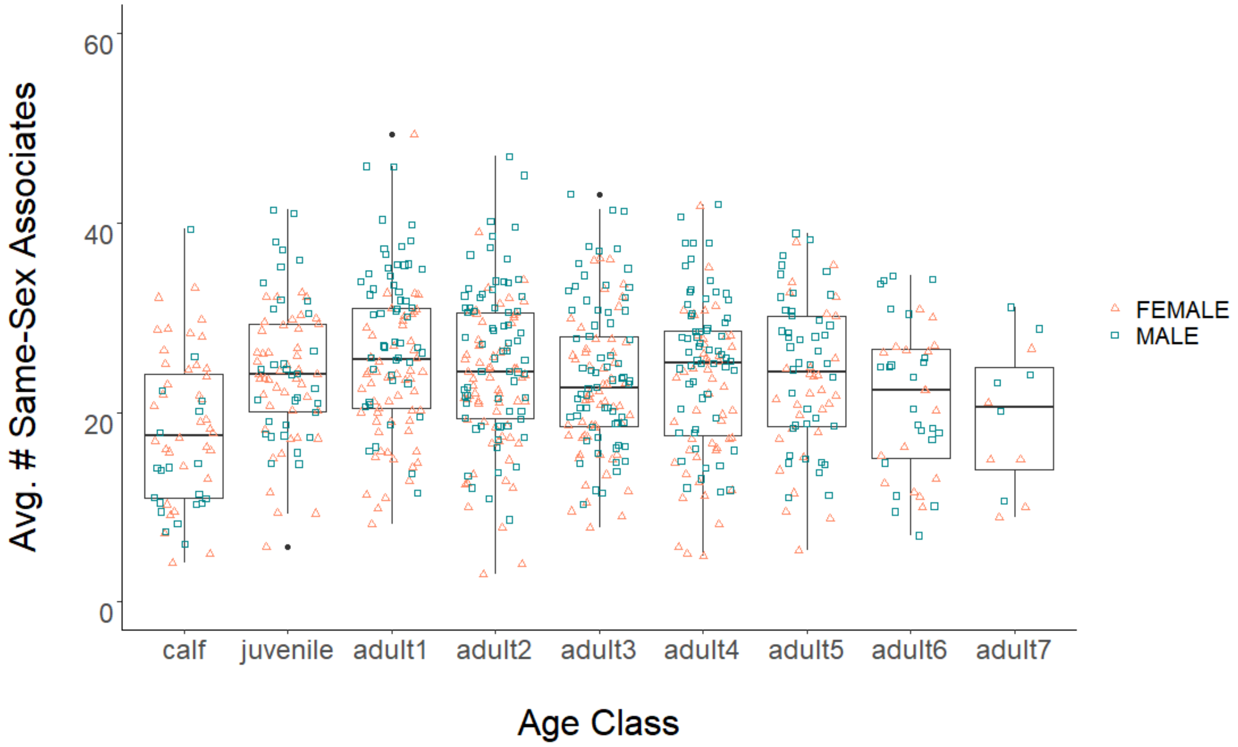


**Supplementary Figure 2.** Phenotypic correlations (Kendall's tau) of the four repeatable social measurements (time alone, in large groups ≥ 7 dolphins, average number of associates and same-sex associates). All correlations were significant (p<0.001).

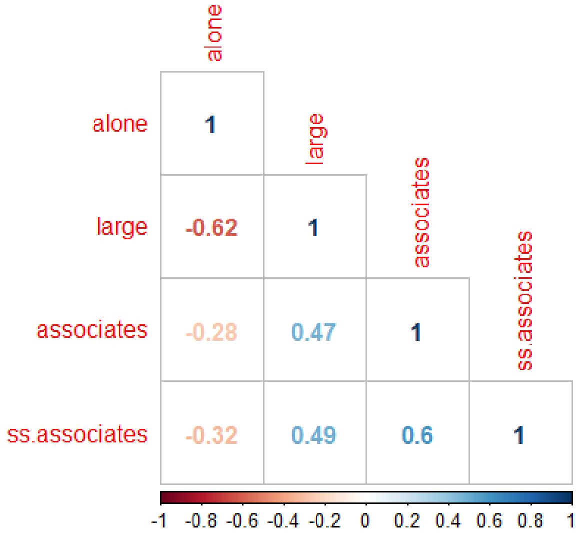

**Supplementary Table 1**. Number time blocks per dolphin in study

| Number of time blocks | Number of dolphins |
|---|---|
| 3 | 70 |
| 4 | 57 |
| 5 | 26 |
| 6 | 19 |
| 7 | 7 |

**Supplementary Table 2**. Dolphins per age category

| Age category | Number of dolphins |
|---|---|
| Calf (0 – weaning) | 56 (36 female, 20 male) |
| Juvenile (weaning – 10 years) | 78 (45 female, 33 male) |
| Adult 1 (10-15 years) | 108 (57 female, 51 male) |
| Adult 2 (15-20 years) | 134 (66 female, 68 male) |
| Adult 3 (20-25 years) | 122 (56 female, 66 male) |
| Adult 4 (25-30 years) | 107 (47 female, 60 male |
| Adult 5 (30 – 35 years) | 72 (30 female, 42 male) |
| Adult 6 (35 – 40 years) | 40 (17 female, 23 male) |
| Adult 7 (45 – 50 years) | 12 (6 female, 6 male) |
| Adult 8 (50+ years) | 2 (2 female) excluded from analyses |
