# Supplementary material for: Lifetime stability of social traits in bottlenose dolphins
Source: Commun Biol. 2021 Jun 18;4:759. doi: 10.1038/s42003-021-02292-x (PMC8213821; doi:10.1038/s42003-021-02292-x)
Supplement: Supplementary file 3 — Reporting Summary [file 42003_2021_2292_MOESM3_ESM.pdf]

nature research

Corresponding author(s): Taylor Evans

Last updated by author(s): May 18, 2021

Reporting Summary

Nature Research wishes to improve the reproducibility of the work that we publish. This form provides structure for consistency and transparency in reporting. For further information on Nature Research policies, see our Editorial Policies and the Editorial Policy Checklist.

Statistics

For all statistical analyses, confirm that the following items are present in the figure legend, table legend, main text, or Methods section.

n/a	Confirmed	
☐	☒	The exact sample size (*n*) for each experimental group/condition, given as a discrete number and unit of measurement
☐	☒	A statement on whether measurements were taken from distinct samples or whether the same sample was measured repeatedly
☐	☒	The statistical test(s) used AND whether they are one- or two-sided<br>*Only common tests should be described solely by name; describe more complex techniques in the Methods section.*
☐	☒	A description of all covariates tested
☐	☒	A description of any assumptions or corrections, such as tests of normality and adjustment for multiple comparisons
☐	☒	A full description of the statistical parameters including central tendency (e.g. means) or other basic estimates (e.g. regression coefficient) AND variation (e.g. standard deviation) or associated estimates of uncertainty (e.g. confidence intervals)
☐	☒	For null hypothesis testing, the test statistic (e.g. *F*, *t*, *r*) with confidence intervals, effect sizes, degrees of freedom and *P* value noted<br>*Give P values as exact values whenever suitable.*
☐	☒	For Bayesian analysis, information on the choice of priors and Markov chain Monte Carlo settings
☒	☐	For hierarchical and complex designs, identification of the appropriate level for tests and full reporting of outcomes
☒	☐	Estimates of effect sizes (e.g. Cohen's *d*, Pearson's *r*), indicating how they were calculated

Our web collection on statistics for biologists contains articles on many of the points above.

Software and code

Policy information about availability of computer code

Data collection	Boat-based observational data were collected as part of a long term study in Shark Bay, Western Australia.
Data analysis	All analyses were conducted using R (version 4.0.2), and specifically the MCMCglmm package.

For manuscripts utilizing custom algorithms or software that are central to the research but not yet described in published literature, software must be made available to editors and reviewers. We strongly encourage code deposition in a community repository (e.g. GitHub). See the Nature Research guidelines for submitting code & software for further information.

Data

Policy information about availability of data

All manuscripts must include a data availability statement. This statement should provide the following information, where applicable:
- Accession codes, unique identifiers, or web links for publicly available datasets
- A list of figures that have associated raw data
- A description of any restrictions on data availability

Data are freely available through Open Science Framework. DOI 10.17605/OSF.IO/RSC9T

Field-specific reporting

Ecological, evolutionary & environmental sciences study design

All studies must disclose on these points even when the disclosure is negative.

Study description	In order to maximize sample size, this study utilized an unbalanced design for longitudinal data. The lifespan was divided into nine time blocks: calf, juvenile, and adulthood, broken into five year blocks from 10 years up until age 50 (there were only two dolphins with sufficient surveys past age 50).
Research sample	179 free-ranging dolphins in the Eastern gulf of Shark Bay met the data requirements (15 or more sightings in at least 3 consecutive time blocks) to be included, 89 females and 90 males. This dataset included 40,523 individual dolphin observations (mean per individual = 377, min 59, max 906).
Sampling strategy	Dolphins were included if they were sighted at least 15 times within three consecutive time blocks. This was sufficient for social metric calculations to stabilize. Having at least three points (i.e. time blocks) per individual is essential fo rrepeatability calculations.
Data collection	This study drew from 67,851 boat-based dolphin observations collected through the Shark Bay Dolphin Research Project (SBDRP) between 1988 and 2019. Researchers with the SBDRP have collected behavioral, demographic, genetic, and ecological data on >1800 Indo-Pacific bottlenose dolphins (Tursiops aduncus) in Shark Bay, Western Australia since 1984.
Timing and spatial scale	Data were collected between 1988 and 2019 as part of the long term research project. Data collection is weather dependent on a daily basis. Researchers generally collect data outside of the windy season (roughly Jan - Apr) on a yearly basis.
Data exclusions	If individuals were sighted multiple times in a day, only the last survey in which it was sighted was included in order to reduce spatial and temporal autocorrelation.
Reproducibility	Data were observational; no experimental controls were involved.
Randomization	In order to account for uneven sampling, we drew a random subsample of 15 surveys per individual's life history stage and calculated seven social measurements. This was repeated 1000x to create an average value per measurement for each individual.
Blinding	Blinding was not relevant, as dolphin ID was the focal point of the analyses. Researchers were trained by JM to have agreement in behavioral calls.

Did the study involve field work? ☒ Yes ☐ No

Field work, collection and transport

Field conditions	Boat based observations were carried out if conditions allowed; generally a beaufort 3 or less, and with little to no active precipitation.
Location	The study site covers roughly 500km2 in the eastern gulf of Shark Bay, offshore from Monkey Mia (25° 47'S, 113° 43'E). Habitat consists of embayment plains (5–13 m), shallow sand flats (0.5–4 m), seagrass beds (0.5–4 m), and bisecting deep channels (7–13 m) (Patterson & Mann 2011).
Access & import/export	Researchers were based at the Monkey Mia Resort, which has a boat launch. Research was conducted under Georgetown University Animal Care and Use permits: IACUC-13-069, 07-041, 10-023 and 2016-1235; and Department of Parks and Wildlife Permits (Western Australia): SF-009876, SF-010347, SF-008076, SF009311, and SF007457.
Disturbance	Caution was taken to approach dolphins from a lateral position at low speed, and to maintain a safe distance while following. When possible the boat was kept in neutral or with engines off.

Reporting for specific materials, systems and methods

We require information from authors about some types of materials, experimental systems and methods used in many studies. Here, indicate whether each material, system or method listed is relevant to your study. If you are not sure if a list item applies to your research, read the appropriate section before selecting a response.

Materials & experimental systems

n/a	Involved in the study	
☒	☐	Antibodies
☒	☐	Eukaryotic cell lines
☒	☐	Palaeontology and archaeology
☐	☒	Animals and other organisms
☒	☐	Human research participants
☒	☐	Clinical data
☒	☐	Dual use research of concern

Methods

n/a	Involved in the study	
☒	☐	ChIP-seq
☒	☐	Flow cytometry
☒	☐	MRI-based neuroimaging

Animals and other organisms

Policy information about studies involving animals; ARRIVE guidelines recommended for reporting animal research

Laboratory animals	This study did not involve laboratory animals.
Wild animals	Wild Indo-Pacific dolphins (Tursiops aduncus) were observed over 32 years. This was an observational study; no animals were removed from the environment. All demographic groups were covered, both males and females and of all age classes.
Field-collected samples	This study did not involve sample colletion.
Ethics oversight	Research was conducted under Georgetown University Animal Care and Use permits: IACUC-13-069, 07-041, 10-023 and 2016-1235; and Department of Parks and Wildlife Permits (Western Australia): SF-009876, SF-010347, SF-008076, SF009311, and SF007457.

Note that full information on the approval of the study protocol must also be provided in the manuscript.
